# Health-related quality of life after otologic surgical treatment for chronic otitis media: systematic review

**DOI:** 10.3389/fneur.2023.1268785

**Published:** 2023-11-02

**Authors:** Esther M. M. Schouwenaar, Catharine A. Hellingman, Jérôme J. Waterval

**Affiliations:** ^1^Department of Otorhinolaryngology and Head and Neck Surgery, Maastricht University Medical Center, Maastricht, Netherlands; ^2^School for Mental Health and Neuroscience, Maastricht University Medical Center, Maastricht, Netherlands

**Keywords:** chronic otitis media, HRQoL, cholesteatoma, otologic surgery, PROM

## Abstract

**Objective:**

This systematic review aims to describe the impact of otologic surgery as a treatment for chronic otitis media (COM) on the Health-Related Quality of Life (HRQoL) of adult patients.

**Methods:**

A literature search was performed in PubMed, Scopus, Embase, and Web of Science until May 2023. Prospective studies including adult patients with COM (cholesteatoma) who underwent canal wall up mastoidectomy, canal wall down mastoidectomy, or tympanoplasty without mastoidectomy, with pre- and postoperative HRQoL measurements, were considered eligible. Questionnaire validation studies were excluded. The risk of bias and study quality were evaluated with a Quality Assessment Tool (for before-after studies with no control group). To assess the change in HRQoL, pre- and postoperative HRQoL values and absolute changes were extracted, synthesized, and presented in tables. Standardized mean differences (SMD) were calculated to enhance comparisons.

**Results:**

Of the 720 studies identified, 16 met the inclusion criteria of this review. Different questionnaires were used throughout the studies. The CES and COMOT-15 were used in five studies and the ZCMEI-21 and COMQ-12 in three studies. All studies indicated statistically significant improvement in HRQoL from pre- to postoperative, measured with disease-specific HRQoL questionnaires. General HRQoL questionnaires did not show significant improvement. Calculated SMDs ranged from 0.24 to 6.99.

**Discussion and conclusion:**

Included studies had low (*n* = 10) to high (*n* = 6) risk of bias and poor (*n* = 4), fair (*n* = 7) or good (*n* = 5) study quality. Surgical treatment positively impacts the HRQoL of adult COM patients with and without cholesteatoma. However, the clinical relevance of the reported changes is unknown due to the lack of minimal clinically important differences (MCID) or cut-off values in each questionnaire. Therefore, further research regarding the MCIDs of each questionnaire is needed. Future research should also report preoperative chief symptoms and indications for surgery to improve individual patient counseling.

## 1. Introduction

Chronic otitis media (COM) is a common infectious and/or inflammatory disease with an annual incidence rate of ~4.76% worldwide (1). It is an important preventable and treatable cause of hearing loss (2). Different definitions of COM are used in literature. According to the World Health Organization (WHO), COM is a persistent inflammation of the middle ear and/or mastoid cavity characterized by chronic (at least 2 weeks) or intermittent otorrhea, tympanic membrane perforation, otalgia, ear discomfort and hearing loss (2). Another reported definition is a disease of the middle ear and/or mastoid with irreversible mucosal damage or infection lasting more than 3 months and characterized by a slow progression of symptoms including recurrent infection and hearing loss (3). Furthermore, a variety of terms are used to categorize COM. Nowadays, COM is categorized into chronic suppurative otitis media (CSOM) and COM with cholesteatoma development (4). Another categorization is squamous or mucosal COM, which can be active or inactive. In this categorization, active squamous COM indicates cholesteatoma, inactive squamous means a retraction (pocket)/atelectasis, active mucosal COM suggests a pathology with discharge through a tympanic membrane perforation, and inactive mucosal COM suggests a dry perforation (5). This diversity in terms and definitions indicates a heterogenous study population that should be considered when performing research on this topic and upon interpretation of performed studies. We will refer to the total COM population, consisting of all these terms, as ***COM w/wo*** ***cholesteatoma***.

Acquired cholesteatoma is a keratinocyte hyperproliferation disorder with sustained keratin desquamation resulting in a cyst-like mass formation from the tympanic membrane into the middle ear and mastoid (6). Cholesteatoma by itself is associated with infectious otitis media which results in osteitis and bony erosion of surrounding structures including the ossicular chain and otic capsule (7). Cholesteatoma can thereby cause hearing loss, vestibular dysfunction (7), and potentially dangerous intracranial complications, such as meningitis, encephalitis, or facial nerve paralysis (8). COM is reported to seriously impact daily life due to hearing impairment, corresponding difficulties in communication, recurrent otorrhea, and frequent doctor visits (9). Social stigmatization associated with persistent ear discharge, ear pain, and the need to avoid water further interferes with social interactions (10). This impediment in professional and social interactions makes COM a serious disabling disease in the otolaryngologic field (9).

### 1.1. Treatment

Surgery for COM and cholesteatoma has the primary goal of completely eradicating the disease and creating a safe and dry (middle) ear (11). In the case of cholesteatoma, surgery is the primary treatment option to prevent further bone destruction and intracranial complications (12). In contrast, treatment of COM without cholesteatoma is not necessarily surgical. Infectious and inflammatory pathology primarily consists of conservative treatment with topical or systemic medication and ear cleaning with microsuction. Surgery for a retraction pocket, atelectasis, or asymptomatic tympanic membrane perforation is performed in consultation with the patient, after weighing the pros and cons, and primarily based on the burden of symptoms. Furthermore, cholesteatoma surgery is indicated even in the absence of complaints or symptoms due to the risk of complications. Various surgical techniques are used in COM, with canal wall up mastoidectomy (CWU), canal wall down mastoidectomy (CWD), and tympanoplasty (without mastoidectomy) being the most common ones, hereafter referred to as ***otologic surgical*** ***treatment***.

### 1.2. Health-related quality of life and aim of the study

Evidence about the effectiveness and safety of surgical treatment in COM has always primarily been focused on changes in objective measurements, such as complete eradication of disease, audiometric outcomes, complications and recurrence rates, and integrity of the tympanic membrane (13–15). However, these objective measures do not necessarily correspond with the patients' personal experiences. For instance, audiometric data were found to correlate with HRQoL outcomes whereas having a dry ear did not (8, 16). Patient-reported symptom appraisal together with results of objective measurements are needed to assess the results of otologic surgical treatment (17). In recent years, quality of life (QoL) has gained interest in healthcare and in the field of otolaryngology to assess the impact of disease (18) and the appreciation of surgical treatment results (17). Health-related quality of life (HRQoL) is a subset of QoL that explicitly reflects how disease influences the wellbeing of a person in the physical, mental, and social spheres of life (19). Therefore, HRQoL measurements become increasingly important as an outcome of middle ear surgery (20) as these reflect the overall impact of the disease and treatment from a patient's perspective instead of the clinician's view.

In recent years, several disease-specific questionnaires to measure HRQoL in patients with COM have been developed, validated, and translated. These include the Chronic Otitis Media Questionnaire (COMQ-12) (21), Chronic Ear Survey (CES) (3), Zurich Chronic Middle Ear Inventory (ZCMEI-21) (22), and Chronic Otitis Media Outcome Test (COMOT-15) (23). All questionnaires have a slightly other focus, as can be seen in **Table 2**. Studies that assess the impact of surgical treatment on the HRQoL of COM patients are increasing. Recently, a systematic review (24) focused on the available instruments to measure HRQoL in COM patients, the timing of the postoperative measurement, and the comparison between different surgical techniques. However, there is no comprehensive overview that objectifies the impact of otologic surgical treatments for COM on the patients' HRQoL, measured exclusively prospectively. Therefore, the aim of this systematic review is to assess the impact of otologic surgical treatment for chronic otitis media with or without cholesteatoma on the health-related quality of life of adult patients. To achieve this aim, studies with at least one preoperative and postoperative HRQoL measurement moment are required to gain knowledge about the HRQoL change from pre- to postoperative.

## 2. Methods

This systematic review was performed according to the guidelines of the Preferred Reporting Items for Systematic Reviews and Meta-Analyses (PRISMA) (25). The protocol for this study is not registered.

### 2.1. Eligibility criteria

The elaborated eligibility criteria can be found in Supplementary Table 1. All original studies with a prospective design written in English or Dutch were included. Conference abstracts, letters, reviews, editorials, and translation and validation studies of questionnaires were excluded. Only studies with adult (≥18 years) patients suffering from chronic otitis media with or without cholesteatoma who underwent otologic surgical treatment were included. As stated in the introduction, various terms to subdivide COM are used in literature. We decided to include all these terms and therefore this systematic review included a heterogenous group of COM patients. To simply refer to this total population, we used the umbrella term ***COM with or without (w/wo)*** ***cholesteatoma*** throughout this review.

Radical cavity reconstruction surgery, subtotal petrosectomy, and grommets insertion were excluded. Canal wall up mastoidectomy (CWU), canal wall down mastoidectomy (CWD), and tympanoplasty without mastoidectomy were included. These surgical techniques together were termed ***otologic surgical treatment*** throughout this review.

Only studies that measured HRQoL pre- and postoperatively with a validated questionnaire were included.

### 2.2. Data sources and search strategy

Studies published until 18 May 2023 were searched using PubMed, Web of Science, Embase, and Scopus. All four databases were searched with a search string based on the eligibility criteria, without any filters or restrictions applied. The universal search string was composed of “Chronic Otitis Media” OR “Cholesteatoma” (Population), “Quality of Life” (Outcome), and “surgical intervention” (Intervention). An elaborated search string per database is given in Supplementary Table 2. Additionally, a manual search of reference lists of eligible articles was performed.

### 2.3. Study selection

Retrieved publications were imported into Rayyan to identify and remove duplicates. Two independent reviewers (ES, JW) first screened the title and abstract to identify potentially relevant records and subsequently, the full-text papers were assessed. Publications that did not meet the predefined eligibility criteria were excluded. The two researchers discussed inconsistencies to reach a consensus.

### 2.4. Quality and risk of bias assessment

Quality assessment to identify the risk of bias for each study was performed by two independent researchers (ES, JW) according to the study quality assessment tool for before-after (pre-post) studies with no control group from the National Heart, Lung, and Blood Institute (NHLBI) (26). This checklist elicits yes/no/not reported/not applicable/cannot determine answers on 13 items with different topics including the methodology, design, and reporting of the study, shown in Supplementary Table 3. Based on the information gathered with this assessment tool, an overall risk of bias judgment and study quality assessment was made. It is important to note that the list of items is not designed to act as a quantitative judgment about the study's quality. The judgment depends on the reviewers' critical appraisal of each item. Inconsistencies between the reviewers were discussed to reach a consensus.

### 2.5. Data extraction

Data were collected independently by both reviewers in a predetermined Excel sheet. Study characteristics and relevant data to answer the research question of the systematic review were extracted from the studies. The data abstraction forms created in Excel were based on the “Checklist of items to consider in data collection or data extraction” (27).

Extracted details were authors and date, study aims, study design, setting, sample size, inclusion/exclusion criteria, participant characteristics, definition of the disease (chronic otitis media, cholesteatoma), HRQoL measurement moments, HRQoL measurement instrument, performed otologic surgical treatment, follow-up period, statistical methods of data analyses, power calculation, outcome values of HRQoL questionnaires, outcomes of statistical analyses of HRQoL questionnaire, loss to follow-up, handling of missing data, limitations reported by the authors, and interpretation of the findings. Finally, for the HRQoL questionnaire outcomes, the means or medians and standard deviations or interquartile range and *p*-values were extracted where possible.

### 2.6. Effect measures

The outcome measure of interest of this systematic review was the difference in HRQoL measured with a validated questionnaire before and after surgery for chronic otitis media w/wo cholesteatoma. No cut-off points were available in the literature for the questionnaires to quantify the reported HRQoL. As a consequence, this review focuses on published pre- and postoperative mean or median values of overall HRQoL scores with standard deviations (SD) or interquartile ranges (IQR), respectively; and *p*-values of the mean differences between the pre- and postoperative HRQoL scores.

The standardized mean differences (SMD) were calculated as the effect measure. Data pooling with pooled effect estimates is desirable, however, this was not possible due to the heterogeneity within the HRQoL questionnaires used and the non-uniform reporting of the outcome of interest. Furthermore, not all studies were suitable for the calculation of these effect measures, leading to a more qualitative and descriptive appreciation of the available data.

SMDs and their precision (95% CI) were calculated with the “Campbell collaboration effect size calculator” (28) based on the reported mean and SDs. In cases in which median and IQR were reported, a web-based calculator (29) was used to calculate estimated means and SDs. The SMD was interpreted as follows: SMD 0.2-0.5 small effect, 0.5–0.8 medium effect, >0.8 large effect (30). When only the mean differences between the pre- and postoperative values were reported in the study, these values and the *p-*value were used to interpret the results.

### 2.7. Data synthesis

Relevant data from each individual study are represented in several tables, Table 1: Study characteristics, Table 2: Characteristics of HRQoL questionnaires used, and Table 3: Individual study results, to enhance comparison. A descriptive explanation of the key findings is provided to emphasize their importance.

**Table 1:** Study characteristics of included studies.

**References**	**Country**	**Study design**	**No. of participants**	**Participant characteristics *COM status, mean age (±SD) or median age (range)***	**Surgery technique**	**Questionnaires**	**Measurement moments *Preoperative (pre); postoperative(post)***	**Additional information**
Baumann et al. (8)	Germany	Prospective study	90	- Patients with CSOM with or without cholesteatoma - Median age 48 years (18–75)	Tympanoplasty, CWU or CWD	SF-36, COMOT-15	Pre: NR Post: 6 and 12 months	CWU or CWD was performed in case of cholesteatoma.
Lailach et al. (31)	Germany	Prospective clinical case study	102	- Patients with chronic mesotympanic otitis media (*n =* 62) and patients with cholesteatoma (*n =* 40) - Mean age 49.31 years (16.04)	Intact canal wall (*n =* 84), CWD (*n =* 18)	SF-36 COMOT-15 ZCMEI-21	Pre: 1 day Post: 6 months	Only graphical representation in bar charts of QoL values without reporting exact values.
Nallapaneni et al. (32)	India	Prospective analytical comparative cohort study	75	- Patients with unilateral inactive mucosal COM - Mean age 33.12 year (8.1)	Tympanoplasty type 1 (*n =* 27), 2 (*n =* 39), or 3 (*n =* 9) with or without cortical mastoidectomy	COMOT-15	Pre: NR Post: 6 months	Type of procedure decided per-operatively Clustered patients based on MERI scores in three groups (mild, moderate, severe) and performed the analyses within these groups.
Cavaliere et al. (33)	Italy	Observational retrospective study with prospectively gathered QoL data	52	- Patients with monolateral COM with (*n =* 38) or without (*n =* 14) cholesteatoma lasting more than 6 months. - Mean age 48.3 years (16.1)	CWDT (*n =* 31) CWUT (*n =* 7) U-MPL (*n =* 12) O-MPL (*n =* 2)	COMOT-15	Pre: NR Post: 12 months	CWUT, U-MPL and O-MPL were taken together as closed techniques vs. CWDT as open technique.
Nurmukhamedova et al. (34)	Uzbekistan	Prospective observational study	60	- Patients with chronic purulent otitis media - Mean age 39.12 years (8.2)	Tympanoplasty, not further specified	COMOT-15	Pre: NR Post: 12 months	No clear description of diagnosis and performed surgery. Result tables unclear, no standard deviation given.
Choi et al. (35)	Korea	Prospective questionnaire-based outcome study.	156	- Patients with COM with (n = 44) or without (n = 112) cholesteatoma - Mean age 50.5 years (IQR 42.1–59.0 years) - Revision (*n =* 26) and primary (*n =* 130) surgery	Tympanoplasty without mastoidectomy (*n =* 52), CWU mastoidectomy (*n =* 72), CWD mastoidectomy (*n =* 34)	CES	Pre: NR Post: 12 months	Compared patients with and without cholesteatoma in the analyses
Nadol et al. (3)	United States	Prospective longitudinal study	93	- Patients with active and inactive COM with or without cholesteatoma - Mean age 44.3 years (16)	Surgical intervention, not further specified	CES	Pre: NR Post: 6 and 12 months	93 patients completed the 6 months follow-up and 73 patients the 12 months follow-up. Only graphical representation in bar charts of HRQoL. No p-values or relevant effect measures reported. No surgical intervention specified
Jung et al. (36)	South-Korea	Prospective questionnaire-based study outcome	41	- Patients with COM with (*n =* 31) and without cholesteatoma (*n =* 10) - Mean age 47.3 years (10.1) - Primary (*n =* 21) and revision (*n =* 20) surgery	CWD mastoidectomy (*n =* 33), CWU mastoidectomy (*n =* 8)	CES	Pre: NR Post: 12 months	Specifically compared primary (*n =* 21) and revision (*n =* 20) surgery group.
Lucidi et al. (37)	Italy	Prospective observational study	85	- Patients with COM with (*n =* 50) and without (*n =* 35) cholesteatoma - Mean age 44 years (14) - Primary (*n =* 64) and revision (*n =* 21) surgery	Endoscopic tympanoplasty	CES	Pre: within 1 month Post: on average 14.9 months (±6)	Endoscopic surgery DASS-21 questionnaire used for depression, stress and anxiety. Compared patients with and without cholesteatoma in the analyses
Nair et al. (38)	India	Prospective observational study	32	- Patients with middle ear cholesteatoma - Median age 37 years (15–69)	TEES: Endoscopic CWU mastoidectomy (*n =* 28) Endoscopic CWD mastoidectomy (*n =* 4)	CES SF-12V2	CES: pre NR, post 6 months SF-12V2: Pre NR, Post 1 month	Solely cholesteatoma patients One patient <18 years old One patient was recurrence case. Only transcanal endoscopic ear surgery
Bächinger et al. (39)	Germany	Prospective longitudinal study	103	- Patients with COM with (*n =* 25) or without cholesteatoma (*n =* 17), or recurrent disease (*n =* 39) - Mean age 51.0 years (15.7)	Tympanomastoid surgery, not further specified	ZCMEI-21	Pre: NR Post: 6 months	12 patients had hearing restoration and 10 patients had open mastoid cavity reduction as indication for the surgical intervention.
Bächinger et al. (40)	Germany	Prospective longitudinal study	108	- Patients with CMED: COM with cholesteatoma (*n =* 46), COM without cholesteatoma (*n =* 22), persistent mastoid cavity with and without cholesteatoma (*n =* 25), other CMED (*n =* 15). - Mean age 51 years (15.9)	Tympanomastoid surgery, not further specified	ZMCEI-21	Pre: NR Post: 6 months	68/108 patients included with COM as indication for surgery. Compared patients with and without cholesteatoma in the analyses
Weiss et al. (41)	Switzerland and Germany	Prospective observational study	87	- Patients with primary or recurrent cholesteatoma, not further specified - Mean age 45.2 years (16.2)	Middle ear surgery, not further specified.	ZCMEI-21	Pre: NR Post: average 203 days	No standardized postoperative measurement moment. 87 patients were included, postoperative HRQoL data was available for 54 patients. Investigates the association with the ChOLE classification.
Tailor et al. (42)	England	Prospective correlational study	52	- Patients with active COM with (*n =* 37) or without cholesteatoma (*n =* 15) - Mean age 47.3 years (18.3) - Primary (*n =* 35) and revision (*n =* 17) surgery	Tympanoplasty via postaural (*n =* 30), endaural (*n =* 21), and permeatal (*n =* 1) approaches with (*n =* 36) or without (*n =* 16) mastoidectomy	COMQ-12, Euro-QoL-5D-5L, HHIA	Pre: NR Post: 12 months	42 of the 52 enrolled patients returned both baseline and 12-month postoperative COMQ-12 questionnaire.
Bukurov et al. (43)	NR	Prospective observational pretreatment/posttreatment study	167	- Patients with inactive mucosal and squamous COM (*n =* 39), active mucosal (*n =* 49), or active squamous COM (*n =* 79)	Tympanoplasty (*n =* 35), intact canal wall tympanomastoidectomy (*n =* 84), and CWD tympanomastoidectomy (*n =* 48)	COMQ-12 SF-36	Pre: NR Post: 6 and 12 months	No age and gender reported. 145 patients completed 6 months follow-up and 114 patients 12 months follow-up. No raw values of pre- and QoL postoperative measurement reported, only SRM of the improvement. Performed bias adjustment to the outcomes.
Baetens et al. (44)	Belgium	Retrospective analysis of prospective gathered COMQ-12 data	26	- Patients with COM with cholesteatoma - Mean age 35.7 years (20.6)	CWU-BOT	COMQ-12	Pre: NR Post: on average 2.35 (SD 0.64) years	No standard measurement moments indicated.

Study design, study population and surgery technique are reported as stated in the study. Number of participants equals the participants taken into data analyses. Exceptions are reported in the column “additional information.” Patient characteristics are reported as stated in the study; not all studies reported the same characteristics which explains the difference in the table. NR, Not Reported; COM, Chronic Otitis Media; CMED, Chronic Middle Ear Disease; CWU, Canal Wall Up; CWD, Canal Wall Down; MERI, Middle Ear Risk Index; CWU-BOT, Canal Wall Up Bony Obliteration Tympanoplasty; CWUT, Canal Wall Up Tympanoplasty; CWDT, Canal Wall down Tympanoplasty; U-MPL, Underlay Myringoplasty; O-MPL, Overlay Myringoplasty; TEES, Transcanal Endoscopic Ear Surgery; SF-36, Short Form Health Survey 36; COMOT-15, Chronic Otitis Media Outcome Test 15; SF-12V2, Short Form Questionnaire 12 Version 2; ZCMEI-21, Zurich Chronic Middle Ear Inventory 21; CES, Chronic Ear Survey; COMQ-12, Chronic Otitis Media Questionnaire 12; HHIA, Hearing Handicap Inventory for Adults; SRM, Standardized Response Mean.

**Table 2:** Characteristics of included disease-specific HRQoL questionnaires.

**Questionnaire**	**Number of items**	**Score**	**Subscales**						**Recall period**	**MCID**
			**Ear symptoms**	**Hearing function**	**Mental health**	**Health service utilization**	**Lifestyle and work impact / social**	**Others**		
CES *Chronic Ear Survey*	13	0–100 4-/6- point Likert scale	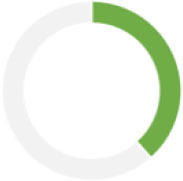	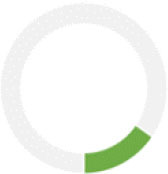	***Absent***	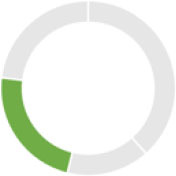	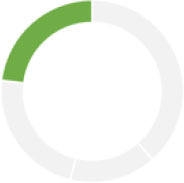		No recall period mentioned, with the exception of:	Unknown
			Ear discharge, pain, odor	Hearing loss		Doctor visits antibiotic use	Water avoidance; Interference with social life		ear drainage and health service utilization 6 months, social impact 4 weeks.	
			Dizziness, tinnitus								
COMOT-15 *Chronic Otitis Media Outcome Test*	15	0–100 6-point Likert scale	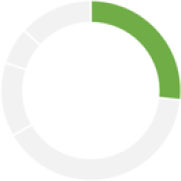	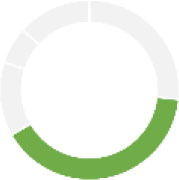	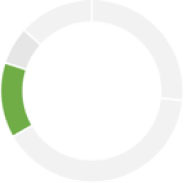	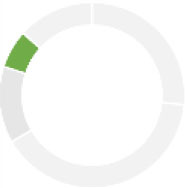	***Absent***	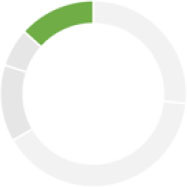	6 months	Unknown
			Ear discharge, pain, pressure, tinnitus	Hearing loss, situational hearing problems	Related to hearing loss	Doctor visits		Overall QoL, headache		
			Odor Dizziness/vertigo							
COMQ-12 *Chronic Otitis Media Questionnaire*	12	0–60 6-point Likert scale	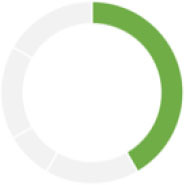	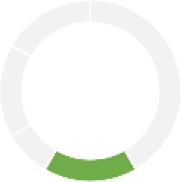	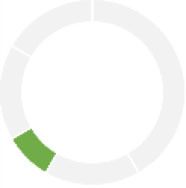	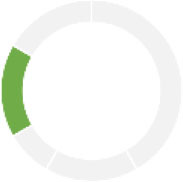	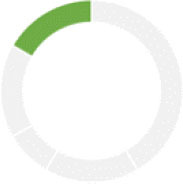		6 months	Unknown values <8 indicate a normal HRQoL in healthy population (44)
			Ear discharge, odor, discomfort, dizziness, tinnitus	Situational hearing problems	Overall impact	Doctor visits, Antibiotic use	Water avoidance, Interference with social/work			
ZCMEI-21 *Zurich Chronic Middle Ear Inventory*	21	0–84 5-point Likert scale	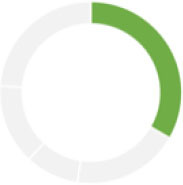	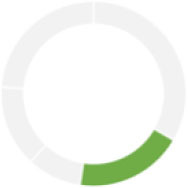	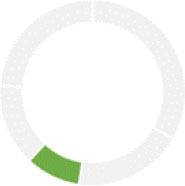	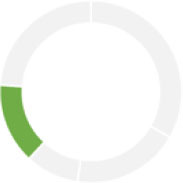	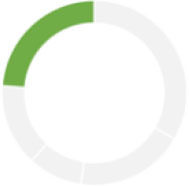		2 weeks health service utilization 6 months	MCID: 5.3
			Ear discharge, Pain, pressure, balance, problems, tinnitus	Hearing loss, Situational hearing problems	Anxiety	Doctor visits, antibiotic use	Water avoidance, interference with social/work			
			Odor							

HRQoL, Health-Related Quality of Life; MCID, Minimal Clinically Important Difference. In all questionnaires higher scores indicate poorer HRQoL. Except for CES in which higher scores indicate better HRQoL. The green part of a circle indicates the portion of the questionnaire which is covered by the specific subscale. All terms to describe symptoms or subscales are abstracted from the questionnaires. Red blocks indicate the absence of a specific factor or whole subscale in the questionnaire.

**Table 3:** Assessment of study results of included studies.

**References**	**HRQoL questionnaire**	**HRQoL questionnaire results Overall mean score (SD) or overall median score (IQR)**			**Difference in HRQoL (summary measure) Preoperative vs. postoperative**		**Conclusion(s)**	**Standardized mean difference (95% CI)**
		**Preoperative**	**Postoperative**		**Preoperative vs. 6 months postoperative**	**Preoperative vs. 12 months postoperative**		
			**6 months**	**12 months**				
Baumann et al. (8)	COMOT-15	46.4 (18.8)	38.4 (20.5)	39.5 (22.0)	*p* = 0.01	*p* = 0.03	Statistically significant improvement in HRQoL from pre- to 6 and 12 months postoperatively in COMOT-15 scores.	0.34 (0.04, 0.63)
	SF-36	NR^*^	NR	NR	NR	NR	Overall mean score not reported. No significant changes in any subscale of the SF-36.	CD
Lailach et al. (31)^**^	COMOT-15	42	33 (13–52)		*p* ≤ 0.001		Statistically significant improvement in HRQoL from pre- to 6 months postoperatively in COMOT-15 and ZCMEI-21 scores. No significant changes in pre- and postoperative scores measured by SF-36.	1.13 (0.84, 1.43)
	SF-36	62	63 (45–86)		*p > 0.05*			0.24 (-0.03, 0.52)
	ZCMEI-21	33 (17–50)	25 (8–41)		*p ≤ 0.001*			1.22 (0.93, 1.52)
Nallapaneni et al. (32)	COMOT-15	47.7 (17.9)	38.9 (17.9)		*p =* 0.01		Statistically significant improvement in HRQoL from pre- to 6 months postoperative COMOT-15 score.	0.49 (0.17, 0.82)
Cavaliere et al. (33)	COMOT-15	36.76 (13.1)		26.88 (12)		*p* = 0.00011	Statistically significant improvement in HRQoL from pre- to 12 months postoperative overall COMOT-15 scores.	0.79 (0.39, 1.19)
Nurmukhamedova et al. (34)	COMOT-15	63.00		18.00		*P* < 0.001	Statistically significant improvement in HRQoL from pre- to 12 months postoperative overall COMOT-15 scores.	CD
Choi et al. (35)	CES	69.1 (53.8–77.1)		92.4 (86.7–96.9)		*p* < 0.001	Statistically significant improvement in HRQoL from pre- to 12 months postoperatively in CES scores.	6.99 (6.41, 7.59)
Nadol et al. (3)	CES	56.9 (17)	70	74.4 (17.9)	NR	NR	Statistically significant improvement in HRQoL from pre- to 6 and 12 months postoperative CES scores. Even statistically significant improvement in QoL between 6 months and 12 months follow-up.	1.00 (0.69, 1.31)
Jung et al. (36)	CES	Primary surgery 44.4 (15.7)		77.3 (17.4)		*p* < 0.001	Both groups showed statistically significant improvement in HRQoL from pre- to 12 months postoperative CES scores. The primary surgery group showed more statistically significant improvement in HRQoL than revision surgery (*p* < 0.03)	1.99 (1.25, 2.72)
	CES	Revision surgery 50.7 (10.0)		73.7 (10.8)		*p* < 0.001		2.21 (1.43, 2.97)
Lucidi et al. (37)	CES	57.8 (18)		71.3 (13) Average follow-up 14.9 months		*p* = 0.000	Statistically significant improvement in HRQoL from pre- to 14.9 months postoperative CES scores.	0.86 (0.55, 1.17)
Nair et al. (38)	CES	44.4 (13.7)	88.3 (4.9)		*p* < 0.001		Statistically significant improvement in HRQoL from pre- to 6 months postoperative overall CES scores.	4.27 (3.38, 5.15)
Bächinger et al. (39)	ZCMEI-21	28.6 (13.6)	21.8 (12.8)		*p < 0.0001*		Statistically significant improvement in HRQoL from pre- to 6 months postoperative ZCMEI-21 scores.	0.51 (0.24, 0.79)
Bächinger et al. (40)	ZCMEI-21	28.8 (13.9)	21.9 (12.9)		*p* < 0.0001		Statistically significant improvement of HRQoL from pre to 6 months postoperative in ZCMEI-21 scores. Even when subgroup analyses were done for CMED, the patients with COM with epitympanic cholesteatoma (*p* = 0.005) and COM without cholesteatoma (*p* = 0.0009) were significant. No statistically significant differences in HRQoL postoperatively between the patient groups.	0.51 (0.24, 0.79)
Weiss et al. (41)	ZCMEI-21	25.1 (15.0)	20.7 (13.2)		*p* = 0.004		Statistically significant improvement in HRQoL from pre- to 6 months postoperative overall ZCMEI-21 score, which was also clinically relevant.	0.31 (-0.07, 0.69)
Tailor et al. (42)	COMQ-12	28.3 (11.6)		14.8 (10.6)		*p* < 0.001	Statistically significant improvement in HRQoL (COMQ-12) and hearing-specific HRQoL (HHIA) scores from pre- to 12 months postoperative. General HRQoL (EQ-5D) was not significantly changed from before to after surgery.	1.21 (0.80, 1.63)
	HHIA	42.9 (28.4)		31.6 (27.5)		*p* = 0.012		0.40 (0.02, 0.79)
	EQ VAS	74.8 (15.9)		78.6 (17.0)		*p* = 0.143		0.23 (-0.15, 0.62)
Bukurov et al. (43)	COMQ-12	NR	NR	NR		SRM: 1.80 (95% CI 1.50;2.10)	Statistically significant improvement in HRQoL from pre- to postoperative COMQ-12 scores. After bias adjustment, the significance remained. However, the generic QoL SF-36 difference did not remain significant after bias adjustment.	NA
	SF-36	NR	NR	NR		SRM: 0.16 (95% CI−0.03; 0.34)		
Baetens et al. (44)	COMQ-12	24.91 (9.48)		9.35 (7.73) Average follow-up 2.35 years		*p* < 0.001	Statistically significant improvement in HRQoL from pre- to 2.35 years postoperative COMQ-12 scores.	1.80 (1.15, 2.44)

^*^Only subscale scores were reported, no overall mean score. ^**^No numerical values were reported, only graphically presented. These are estimated mean scores abstracted from the graphs. If two postoperative measurement moments were present, the mean percentage of change was calculated with the value of the longest follow-up period. SD, Standard Deviation; IQR, Interquartile Range; NR, Not Reported; NA, Not Applicable; COM, chronic otitis media; TPL, tympanoplasty; CWU, canal wall up; CWD, canal wall down; CWU-BOT, canal wall up bony obliteration technique; SF-36, Short Form Health Survey 36; COMOT-15, Chronic Otitis Media Outcome Test 15; ZCMEI-21, Zurich Chronic Middle Ear Inventory 21; CES, Chronic Ear Survey; COMQ-12, Chronic Otitis Media Questionnaire 12; HHIA, Hearing Handicap Inventory for Adults; SRM, Standardized Response Mean; CD, Cannot determine; EQ VAS, EuroQoL Visual Analog Scale; CMED, Chronic Middle Ear Diseases.

## 3. Results

### 3.1. Study selection

**Figure 1:**
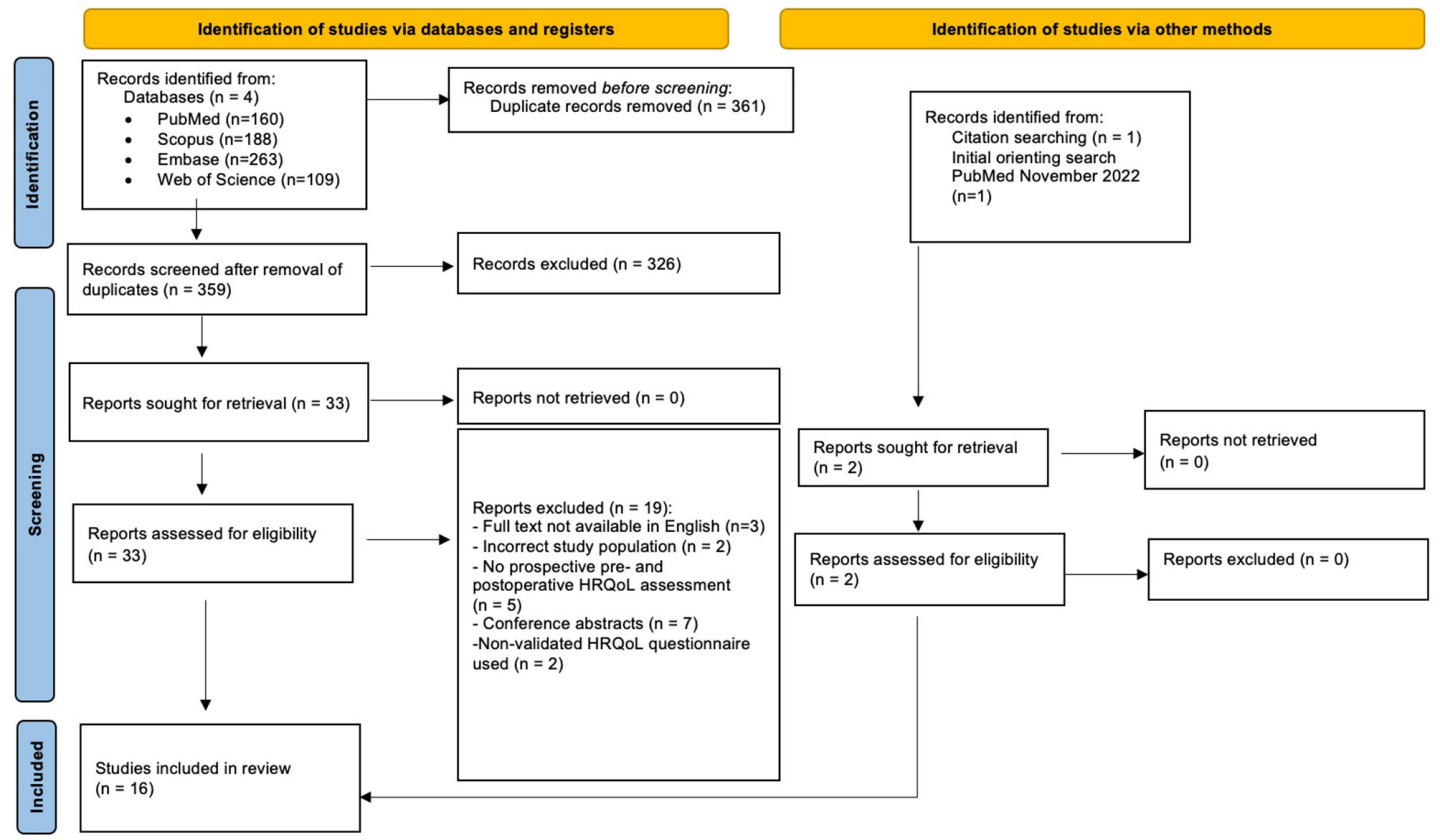
PRISMA 2020 flow diagram.

An indicative search was performed in PubMed in November 2022. The search was updated until May 2023. A total of 720 records were retrieved, of which 361 records were identified as duplicates and removed. Of those remaining 359 records, 326 were excluded upon title and abstract screening. After the full-text assessment of the 33 records that were left, 19 reports were excluded due to several reasons. One report was added manually after reference list screening of the eligible studies (3). Additionally, the initial search yielded one eligible study that could not be retrieved with the updated search (43). Together, this resulted in 16 unique full-text articles (3, 8, 31–44) for inclusion in the systematic review (Figure 1).

### 3.2. Study characteristics

Table 1 represents a detailed overview of the relevant characteristics of the included studies. The studies are clustered based on the HRQoL questionnaire used. All studies were prospective observational studies with pre- and postoperative measurements, published between 2000 and 2023. Information about HRQoL scores before and after otologic surgical treatment was gathered with the use of seven different validated HRQoL questionnaires. The sample sizes per study ranged between 26 (44) and 167 (43) patients, resulting in a total of 1,329 included patients with a mean age of 44.74 years. HRQoL assessment was conducted with at least one disease-specific HRQoL questionnaire in each study. One questionnaire for general chronic middle ear diseases (CES) was used and three COM-specific questionnaires (COMQ-12, ZCMEI-21, and COMOT-15). CES (3, 35–38) and COMOT-15 (8, 31–34) were used in five different studies, and the ZCMEI-21 (31, 39, 40) and COMQ-12 (42–44) in three studies. Furthermore, three general HRQoL questionnaires were used: SF-36 (8, 31, 43), SF-12V2 (38), and Euro-Qol-5D-5L (42). A hearing-specific QoL questionnaire (HHIA) was used once (42). Table 2 provides an overview of the content of each disease-specific HRQoL questionnaire. All questionnaires contain questions on classic ear symptoms, use of medical resources, and impact on daily life activities, except for COMOT-15 which does not encompass the latter. COMOT-15, COMQ-12, and ZCMEI-21 include psychological aspects as well. However, besides covering different domains of HRQoL, each questionnaire has its own interpretation of these domains.

The otologic surgical treatments were described as tympanoplasty (8, 32, 35, 37, 42–44), canal wall up, and canal wall down (8, 31, 35, 36, 43). Five studies did not specify the performed otologic surgical treatment (3, 34, 39–41). Two studies used endoscopic surgery techniques (37, 38). Furthermore, six studies (31, 35–37, 39, 42) reported the distribution of primary and revision surgery in the study population. One study (36) had the same number of participants in the revision and primary surgery group to compare the HRQoL scores. The exact number of patients per surgical technique was not uniformly reported. One study excluded COM patients with cholesteatoma (32), whereas two other studies only included patients with cholesteatoma (38, 41). One study (34) did not specify whether included patients suffered from COM with or without cholesteatoma. The 12 remaining studies included COM patients both with and without cholesteatoma (3, 8, 31, 33, 35–37, 39, 40, 42–44). In the total study population of 1,329 patients, 34% were specified as COM with cholesteatoma, 27% without cholesteatoma, and 39% were not specified.

The actual timing of preoperative measurement was not specified and the follow-up period differed among the included studies. The most common postoperative measurement moments were 6 (31, 32, 38–40) and 12 months (33–36, 42, 43). The COMQ-12 questionnaire was the only one without postoperative measurement at 6 months. The average follow-up period ranged from 6.3 months (ZCMEI-21) to 18 months (COMQ-12).

### 3.3. Quality assessment and risk of bias

With the NHLBI quality assessment tool (26), five out of sixteen studies were graded as “good study quality,” seven as “fair study quality” and the remaining four as “poor study quality” (see Supplementary Table 3). Although not completely corresponding with overall study quality, ten studies were graded as “low risk of bias” and six studies were graded as “high risk of bias.” The risk of recall bias was overall negligible due to the uniform prospective design with pre- and postoperative HRQoL measurements. Lack of information on the context of questionnaire administration and blinding of outcome assessors increased the risk of response bias for all included studies. In three studies (32, 37, 39) the questionnaires were filled in during postoperative consultation in the presence of the doctor which is known to increase the chance of socially desirable answers.

Increased risk of selection bias was present in several studies due to high loss to follow-up (8, 31, 37, 38, 41). The results of the included studies were all exposed to some degree of selection bias, specifically non-response bias or attrition bias, since almost all analyses were performed with data of patients who completed at least the pre- and postoperative questionnaire. Missing data were not imputed but left out of analyses. A strong selection bias was introduced in one study (33) by giving the postoperative questionnaire only to patients with dry ears without signs of inflammation or healing problems after follow-up.

### 3.4. Results of individual studies

In Table 3, a detailed overview of the results for each study is shown. All the studies reported a statistically significant improvement in disease-specific HRQoL scores after otologic surgery among COM w/wo cholesteatoma patients. General HRQoL questionnaires did not detect an impact of surgery among COM patients w/wo cholesteatoma on the HRQoL (8, 31, 42, 43). The significance of improvement in disease-specific HRQoL was independent of the follow-up period.

One study (31) only presented the outcomes of the pre- and postoperative HRQoL measurements graphically which had to be interpreted for this systematic review. Another study (43) reported standardized response means (SRMs) instead of raw values. The SRMs indicated a statistically significant improvement in HRQoL between pre- and postoperative measurements with COMQ-12 and general SF-36. Adjustment of the SRM scores for expected (placebo) bias resulted in smaller SRMs of the COMQ-12 measurements, causing the SRM of the SF-36 values to lose its significance. All reviewed studies published *p-*values with a significance level of *p* < 0.05, except for one study (3) that only described the results as statistically significant improvement without *p*-values or other relevant numbers.

Although not shown in Table 3, four studies (8, 35, 37, 40) assessed the influence of cholesteatoma on the HRQoL change. None indicated a significant difference in HRQoL change between COM with and without cholesteatoma. Two studies (38, 41) included only patients with cholesteatoma which both showed significant improvement in HRQoL after endoscopic surgery (38) and after unspecified middle ear surgery (41). A study (36) that explicitly compared primary surgery (*n* = 21) with revision surgery (*n* = 20) showed greater improvement in HRQoL for the primary surgery group, however, the difference was not significant. None of the included studies directly compared different surgical techniques. One study (33) reported results separately for open and closed techniques, which both showed a significant HRQoL change but without comparison.

Standardized Mean Differences (SMD) were calculated for each study if possible. The small majority, 9/16 SMDs, indicated a large increase in HRQoL after surgery (SMD > 0.8) for COMOT-15 and ZCMEI-21 (31), COMQ-12 (42, 44), and all studies that used the CES questionnaire (3, 35–38). Medium SMD (0.5–0.8) was calculated for the ZCMEI-21 (39, 40) and COMOT-15 (33). A small SMD (<0.5) was found for COMOT-15, SF-36 (31), and ZCMEI-21(41). The SMD was not calculated for two studies (34, 43) due to missing data. Overall, the effect measure indicated a considerable positive impact of otologic surgical treatment on the HRQoL of COM w/wo cholesteatoma patients which is in line with the *p*-values abstracted from the studies.

## 4. Discussion

### 4.1. Summary of evidence

The aim of this systematic review was to investigate the impact of otologic surgical treatment on the health-related quality of life of chronic otitis media patients with or without cholesteatoma. In total, 16 studies were considered eligible and were included in this review. All studies reported a statistically significant HRQoL improvement after otologic surgical treatment compared to before measured with various disease-specific HRQoL questionnaires. Contrarily, studies that used general QoL questionnaires SF-36 (8, 31, 43) and EQ-5D VAS (42) reported no statistically significant improvement after surgical treatment, even when the specific and general QoL questionnaires were administered simultaneously within the same study population. General QoL measurement instruments insufficiently cover disease-specific symptoms or other features that may affect daily life (8, 31, 45) and generic instruments are not responsive to detect changes caused by effective treatment (46). Nonetheless, general QoL tools are important to measure the impact of various diseases on the general QoL and thereby make standardized comparisons possible between different diseases or healthy and diseased populations (8). Disease-specific questionnaires are preferable to investigate the effect of otologic surgical treatment on specific otologic symptoms.

An overall large effect or increase of HRQoL after surgical treatment for COM is suggested by the calculated SMDs. Interestingly, COMOT-15, ZCMEI-21, and SF-36 were used within one study population (*n* = 102) (31). The results showed a large SMD for COMOT-15 and ZCMEI-21, 1.13 and 1.22, respectively, and a small SMD, 0.24, for the general SF-36. These results confirm the difference between disease-specific and general HRQoL questionnaires. This study indicated no difference in the magnitude of HRQoL change measured by the COMOT-15 and ZCMEI-21. The differences in calculated SMDs cannot completely be explained due to the incomplete reporting of and heterogeneity in surgical treatment, study population, and questionnaires used between the various studies.

### 4.2. Questionnaires

Even though all disease-specific HRQoL questionnaires demonstrated statistically significant improvement in several studies, the differences between the questionnaires might partially explain the observed differences in SMDs. The content of each questionnaire is reported in Table 2. All questionnaires have the same content except for CES which does not cover psychological impact and COMOT-15 which does not include impact on daily life activities.

To emphasize, as Table 2 illustrates, ZCMEI-21 and COMQ-12 may be the most complete HRQoL assessments for COM patients presently. The COMQ-12 contains two general questions regarding the impact on daily activities and mental health whereas ZCMEI-21 comprehensively assesses the psychosocial aspect with seven items regarding fear of future problems, social/daily activities, sleep quality, and sadness. However, this extensive assessment increases the number of items of the ZCMEI-21.

Another aspect of questionnaires is the recall period respondents have to report about. COMQ-12 has a long recall period of 6 months whereas ZCMEI-21 has a short recall period of 2 weeks. A long recall period may introduce recall bias, however, a short recall period may not capture all experienced symptoms. No concrete statement is available regarding the best recall period, as is elaborated on in paragraph 5.5 “Timing of questionnaire administration.” In a research setting, ZCMEI-21 may be the most suitable to measure HRQoL among COM patients due to its complete content on each component of HRQoL. In a clinical setting, we would prefer to use COMQ-12 as the patient reported outcome measure (PROM) due to its favorable length and concise and complete content.

As was mentioned before, the CES does not cover psychological and emotional aspects of the perceived impact of COM on the HRQoL while these aspects are an important part of the HRQoL construct. Patients with more prominent depressive symptoms and mood disturbances have significantly higher COMOT-15 and ZCMEI-21 scores and significantly lower SF-36 scores (31), all indicating lower HRQoL. Moreover, hearing loss and the corresponding communication impairment are associated with depression, social withdrawal, and anxiety and thereby limit a patient's HRQoL (47). This incomplete coverage of the HRQoL construct by the CES might explain the high percentages of change and large SMDs reported with CES. One could logically expect a decrease in HRQoL improvement measured by the CES once psychological and emotional aspects are included.

The COMOT-15 is highly focused on hearing level with 7/15 items covering this subject. The degree of hearing impairment is used as an objective measurement to indicate the success of surgical treatment. Hearing loss is known to lower QoL in general (10, 48) and hearing aids are known to increase HRQoL (49, 50). This questions whether COMOT-15 is “overweighted” by hearing-related questions or whether this focus is actually needed for this otologic population. A multinational collaborative study demonstrated that patients with a higher degree of hearing loss had a poorer HRQoL, measured with COMQ-12. Moreover, this study argued postoperative hearing improvement is a better indicator of surgical success from a patient's perspective than a dry ear (16). A positive relation between the changes in audiometric data and the measured HRQoL improvement was also demonstrated in other studies (3, 8, 22, 36). Above all, hearing loss is stated to be the dominant symptom experienced by COM patients (51). However, in another study, the degree of hearing loss was argued to not adequately reflect the experienced disease burden of COM, nor the impact of treatment (52). This is supported by the lower changes in HRQoL reported by the hearing-specific questionnaire HHIA (SMD 0.40) compared to disease-specific COMQ-12 (SMD 1.21). Nevertheless, both questionnaires indicated statistically significant HRQoL improvement (42). Furthermore, ZCMEI-21 indicated differences in HRQoL between different types of chronic middle ear diseases, including COM with and without cholesteatoma, independent of hearing level. These findings imply a considerable impact of other symptoms besides hearing loss, such as ear discharge, vertigo, and tinnitus, on HRQoL (40). In line with this, a comparison of HRQoL measured with COMOT-15 and CES between patients treated with CWD or CWU implied that hearing loss does not necessarily decrease overall QoL (53). Moreover, the mental health subscale of COMOT-15 contains questions solely focused on the impact of hearing loss. Other ear symptoms are argued to have less influence on mental health than hearing impairment (54). Nevertheless, this focus on hearing may partially restrict patients from expressing the impact of any mental health issues related to other ear symptoms on their HRQoL.

In conclusion, although technically hearing is more prominent in the COMOT-15 than in other questionnaires, clinically, it is debatable whether this is an issue. Comparison of COMOT-15 and ZCMEI-21 within the same study population showed a high Cronbach's α (>0.9) of the hearing subscale which may indicate redundancy (51). These questionnaires may thereby cover comparable facets of hearing. Likewise, the COMOT-15 mental health had a high correlation with hearing level compared to the same correlation of ZCMEI-21 (51). Hence, COMOT-15 may be best suitable for research with a focus on hearing and ZCMEI-21 or COMQ-12 may be better suitable for research with a focus on the whole symptom complex of COM and the related HRQoL.

### 4.3. Surgical technique

Although the surgery technique used might have an influence on HRQoL, due to the lack of consistent notation of the number of patients per surgery technique and corresponding results in the included studies, no definite statement of this influence can be given. CWD tympanoplasty is traditionally thought to have a major negative impact on postoperative HRQoL due to the aftermath of frequent outpatient clinic visits for ear cleaning, vertigo episodes with temperature changes in the external auditory canal, the need to avoid water, and discomfort with hearing aids (55). Nonetheless, convincing scientific evidence of impaired HRQoL is non-existent. Various studies investigated the association between CWU and CWD technique and HRQoL with conflicting results. Results in favor of CWU compared to CWD were demonstrated in univariate analysis (35) and at 6 months postoperatively (56). Contrarily, at 12 months postoperative, no difference was observed (53, 57). Presently, obliteration of the mastoid cavity is increasingly used by many surgeons to decrease the impact of the aftermath of CWD techniques. A comparable HRQoL among cholesteatoma patients after CWD tympanoplasty with mastoid obliteration compared to intact canal wall tympanoplasty was demonstrated (55).

Whether the surgery is primary or revision may have an impact as well. Although it was investigated in just one study with a small population, a significant difference in HRQoL improvement was observed in favor of primary surgery (36). Patients undergoing revision surgery are argued to be more accustomed to their symptoms, resulting in a higher preoperative HRQoL and less improvement after surgery.

### 4.4. Chief symptoms and indication for surgery

As argued above, cholesteatoma requires surgical treatment whereas surgery for COM without cholesteatoma is often indicated after failed conservative treatment. Moreover, indication for surgery in cholesteatoma patients is independent of symptoms whereas surgery in COM patients is a patient-centered decision where the experienced symptoms play an important role. Patients with cholesteatoma may experience no or mild symptoms (40) and might be more concerned about the total eradication of the disease by surgery than any functional outcomes. Considering this, we expected to observe a difference in the reported HRQoL change after surgical treatment between COM patients with and without cholesteatoma, with greater improvement in HRQoL among patients without cholesteatoma. In this review, most of the included studies specified the number of COM patients with and without cholesteatoma within their study population. However, separate analyses per group (with vs. without cholesteatoma) on the HRQoL scores were rarely carried out. Four studies (8, 35, 37, 40) performed these analyses and concluded no significant differences between patients with and without cholesteatoma. Furthermore, no significant difference was observed in the respective pre- and postoperative HRQoL scores between these patients (37). This is in line with the results of another study (52) not included in this review due to the questionnaire used. Two studies in this review with only cholesteatoma patients indicated an improvement in HRQoL with a large (38) and small (41) SMD, respectively. The extent of cholesteatoma was not found to be associated with the HRQoL, nor correlated with any symptoms directly (41). In conclusion, a difference in HRQoL between COM with and without cholesteatoma (before and after surgery) is not demonstrated by our review, nor is it thoroughly investigated in existing literature.

Furthermore, COM is an umbrella term with various manifestations, from dry tympanic membrane perforation to a chronic discharging ear with cholesteatoma. Thus, the burden of experienced symptoms in this population will vary, influencing HRQoL and expectations of surgery. However, the included studies did not report the experienced symptoms of COM patients. This impedes insight into, for instance, the proportion of asymptomatic patients who underwent surgery. Five out of the sixteen included studies reported the indication for surgery (39, 40, 42) and/or chief symptoms preoperatively (35, 36, 42). Otorrhea was the most reported chief symptom followed by hearing loss. Furthermore, the current literature is inconsistent about the impact of improved hearing level or a dry ear on HRQoL after surgery. Some studies report hearing loss as the worst tolerable symptom compared to tinnitus or otorrhea among COM patients (54, 57), whereas others argue a recurrent draining ear has the most impact on the HRQoL (3, 41, 58). As reported by Nadol et al. (3) significant differences in HRQoL scores, measured with CES, between different groups of COM patients exist. The smallest and largest change in HRQoL was observed within the inactive COM group and the inactive with frequent reactivation group, respectively. This suggests a greater impact of surgery on HRQoL in patients with preoperative recurrent ear discharge. However, although measured with non-validated questionnaires, significant HRQoL improvement after tympanoplasty type 1 was observed within a COM population with dry tympanic membrane perforation and was associated with improvement in hearing level (59, 60).

To emphasize, no definite conclusions can be drawn without information on the chief symptoms or indication for surgery in the total study population. Accordingly, categorizing the COM population into with or without cholesteatoma insufficiently considers the impact of experienced symptoms or indication for surgery on HRQoL. Preferably, prospective cohort studies should report preoperative chief symptoms and/or indication for surgery to gain useful insight into the patient's experienced symptoms and reason for surgery in addition to the presence or absence of cholesteatoma. Knowledge of the association between surgery indication, preoperative chief symptoms, and HRQoL change is needed to better understand HRQoL in combination with performed surgery, as this would hugely benefit the preoperative counseling of individual COM patients in the future.

### 4.5. Timing of questionnaire administration

A recently published systematic review of the literature (24) indicated 12 months follow-up as the most suitable time point to assess postoperative HRQoL without potential bias due to various healing times after different surgery techniques used. Additionally, a significant increase in HRQoL from 6 to 12 months postoperatively was reported, independent of surgery technique (3). Contrarily, a more recent prospective study (8) demonstrated a stable HRQoL from 6 to 12 months postoperatively among COM w/wo cholesteatoma patients. In the included studies in our review, the timing of postoperative assessment differed which may have contributed to the differences in SMD.

In addition, another important aspect of timing is the recall period or time frame respondents are asked to base their answers on. CES does not have a specified recall period and thereby measures HRQoL at the moment of administration. COMQ-12 and COMOT-15 refer to the previous 6 months and ZCMEI-21 to the previous 2 weeks. A longer recall period may introduce recall bias. A recall period of weeks rather than months is commonly accepted for PROMs measuring HRQoL (61). Recall bias is thereby minimized in ZCMEI-21 whereas it may be introduced in COMQ-12 and COMOT-15.

However, it is important to bear in mind that the disease of interest is a chronic disease. Recently, a study among patients with chronic ear diseases, including COM w/wo cholesteatoma, indicated significantly fewer experienced symptoms measured in the previous 2 weeks compared to the previous 3 or 6 months. No difference between 3 and 6 months was present (62). Therefore, the recall period in HRQoL assessment among chronically diseased patients should be long enough to capture the natural course of the disease and short enough to minimize recall bias (63). Therefore, the optimal recall period in COM patients w/wo cholesteatoma is still not clear, but it is obvious that the different recall periods in the various questionnaires may influence HRQoL scores.

### 4.6. Statistical significance and clinical relevance

Even though the pre- and postoperative questionnaire scores differed significantly in all included studies, this does not immediately implicate a clinically relevant difference in HRQoL. For that reason, the minimal clinically important difference (MCID), defined as the smallest change in outcome that is relevant to the patient (64), is required. This patient-centered outcome is, thus, dependent on the magnitude of improvement in combination with the value patients relate to the change (64). Considering the patient-centered aspect of this systematic review and the aims of the included studies, the MCID should ideally be investigated. However, the MCID was only calculated for the ZCMEI-21 questionnaire (39) at 5.3 points. With this MCID in mind, all studies that used ZCMEI-21 demonstrated that the difference in HRQoL was clinically relevant. The mean changes between the pre- and postoperative measurements were 8 (31), 6.8 (39), and 6.9 (40). The MCID for the CES, COMOT-15, and COMQ-12 is not reported in the literature to date. Hence, the clinical importance of the HRQoL changes after otologic surgical treatment in COM w/wo cholesteatoma patients is largely unknown. However, for the COMQ-12 questionnaire, a cut-off value of 8 was published (65), which means that a total score of 8 or lower indicates a normal HRQoL. The two included studies that used COMQ-12 (42, 44) reported significantly lower postoperative scores of 9.35 after 28 months follow-up (44) and of 14.8 12 months postoperative (42). These values indicate HRQoL changing to almost normal values, however, the clinical relevance of the change is unknown.

### 4.7. Limitations

#### 4.7.1. Study and outcome level

Reasonably, these results cannot be discussed without taking into account the study quality of the included studies. Overall, the study quality was fair with an intermediate to high risk of bias. Sample sizes were rather small to intermediate and sample size calculations were hardly performed. Description of HRQoL questionnaire administration was mostly absent which increases the risk of response bias in the included studies. Additionally, a high risk of selection bias exists among the majority of included studies since only participants with complete data at baseline and postoperative follow-up were taken into data analyses. The included studies varied in the HRQoL measurement instrument used. Furthermore, different definitions or unreported definitions of COM, different follow-up periods, and otologic surgical treatments together made comparison between the studies and a meta-analysis impossible. Besides these limitations, a strong point of the included studies was their prospective design with preoperative and postoperative assessment of HRQoL. Thereby, recall bias was limited in each study and changes in HRQoL due to surgical treatment could be identified.

#### 4.7.2. Review level

The inclusion of only English and Dutch articles could have led to unidentified eligible articles written in other languages, which does not introduce systematic bias (66), however, relevant information might be missed. Nevertheless, the systematic review included 16 studies that assessed the impact of various otologic surgical treatments, with different questionnaires, on the HRQoL of adult COM patients w/wo cholesteatoma.

### 4.8. Implications for practice and research

The ultimate goal is to be able to advise individual COM patients w/wo cholesteatoma regarding their choice of elective otologic surgery in terms of their specific symptom change. To achieve this goal, the next step in future research should be the determination of the MCID of validated disease-specific HRQoL questionnaires to gain knowledge about the clinical relevance of HRQoL changes for the patients. Without this information, statistically significant results lack the power to fully prove the success of surgical interventions from a patient's perspective. Besides this, the literature is still unclear about associations regarding underlying factors that may influence HRQoL, such as hearing loss, otorrhea, and depressive disorders. Therefore, future studies should report preoperative chief symptoms and/or indication for surgery in order to classify patient groups in addition to the presently used classification of the presence or absence of cholesteatoma. Next, corresponding analyses on symptom level might result in insight into the association of surgery and HRQoL with the experienced symptoms. Ideally, this should assist ear, nose, and throat (ENT) doctors in individual patient counseling. More high-quality research with large sample sizes, comparable study populations, and the same HRQoL questionnaire is needed to draw overall conclusions about the impact of otologic surgical treatment on HRQoL in combination with underlying factors. Furthermore, cholesteatoma patients should be investigated as a separate group and compared to COM patients without cholesteatoma to examine any difference in HRQoL. Routine assessment of HRQoL with one questionnaire at the preoperative consult and postoperative evaluations could facilitate efficient data collection in combination with patient-centered care. As argued above, we would recommend COMQ-12 to be used as a PROM in this clinical research setting. In order to be able to inform patients individually, both subjective as well as objective factors are needed.

## 5. Conclusions

In conclusion, this systematic review provides an overview of the evidence that otologic surgical treatment positively impacts HRQoL among adult COM patients w/wo cholesteatoma, measured by various disease-specific HRQoL questionnaires. Firstly, this implies that COM has a substantial influence on daily life. Secondly, this evidence substantiates the importance of HRQoL assessment in clinical practice. However, the minimal clinically important differences of the questionnaires have not been investigated yet, impeding drawing conclusions on clinical relevance. The systematic review included studies with various otologic surgical treatments and different HRQoL measurements. This diversity makes generalizability of the results to adult COM patients w/wo cholesteatoma with indication for otologic surgical treatment cautiously possible. However, it is important to bear in mind that the fair study quality, intermediate risk of bias, and overall high risk of selection bias indicate that actual outcome parameters might be less positive.

## Data availability statement

The original contributions presented in the study are included in the article/Supplementary material, further inquiries can be directed to the corresponding author.

## Author contributions

ES: Conceptualization, Data curation, Investigation, Methodology, Writing—original draft, Writing—review & editing. CH: Supervision, Validation, Writing—review & editing. JW: Conceptualization, Data curation, Methodology, Supervision, Validation, Writing—review & editing.
