# Supplementary material for: Health-related quality of life after otologic surgical treatment for chronic otitis media: systematic review
Source: Front Neurol. 2023 Nov 2;14:1268785. doi: 10.3389/fneur.2023.1268785 (PMC10654635; doi:10.3389/fneur.2023.1268785)
Supplement: Supplementary file 1 [file Table_1.docx]

Supplementary Material

Health-Related Quality of Life after Otologic Surgical Treatment for Chronic Otitis Media: Systematic Review

# Supplementary Tables

Supplementary Table S1: Eligibility criteria

| [Abbreviation: QoL: Quality of Life] | | |
| --- | --- | --- |
|  | **Inclusion** | **Exclusion** |
| **Population** | - Adult patients suffering from chronic otitis media without cholesteatoma  - Adult patients suffering from chronic otitis media with cholesteatoma  - Adult patients suffering from cholesteatoma | - Patients suffering from isolated Eustachian tube dysfunction  - Pediatric study population  - Specific study population with patients suffering from down-syndrome or cleft palate |
| **Intervention** | - Tympanoplasty  - Mastoidectomy  - Mastoid obliteration  - Canal wall up tympanoplasty with or without mastoidectomy  - Canal wall down tympanoplasty with or without mastoidectomy  - Endoscopic ear surgery | - Subtotal petrosectomy  - Ventilation tubes insertion  - Eustachian tuboplasty  - Meatoplasty  - Revision surgery  - Radical mastoidectomy reconstruction |
| **Outcome** | - Pre- and postoperative QoL measured by a validated QoL questionnaire   - Chronic Otitis Media Questionnaire 12 (COMQ-12) - Chronic Ear Survey (CES) - Zurich Chronic Middle Ear Inventory 21 (ZCMEI-21) - Chronic Otitis Media Outcome Test (COMOT-15) | - Non-validated QoL questionnaire  - Adaptation, translation, and validation studies of QoL questionnaires |
| **Study design** | - Prospective studies with pre- and postoperative measurement moment of the quality of life | - Case reports  - Letter to the editor  - Editorials  - Reviews  - Conference abstracts  - Systematic reviews  - Meta-analysis |
| **Language** | - English, Dutch | - Other languages |

Supplementary Table S2: Search string per database

| **Database** | **Search string** |
| --- | --- |
| PubMed | (("Otitis Media" [Mesh] OR "Chronic Otitis Media" [Title/Abstract] OR cholesteatoma [Mesh] OR cholesteatoma [Title/Abstract]) AND ("Quality of Life"[Mesh] OR QoL [Title/Abstract] OR "Health-related quality of life" [Title/Abstract] OR HRQoL [Title/Abstract] OR "Well-being" [Title/Abstract])) AND ((((((((tympanoplasty[MeSH Terms]) OR (Tympanoplasty)) OR ((myringoplasty) OR (myringoplasty[MeSH Terms]))) OR ((mastoidectomy) OR (mastoidectomy[MeSH Terms]))) OR ("Mastoid obliteration" [Title/Abstract] OR "Bony obliteration Tympanoplasty" [Title/Abstract])) OR ("Canal Wall up" [Title/Abstract] OR "Canal Wall Up Tympanoplasty" [tiab])) OR ("Otologic Surgical Procedures"[Mesh] OR "Otologic Surgical Interventions" OR "Surgical Treatment")) OR ("Canal Wall Down" [Title/Abstract] OR "Canal Wall Down Tympanoplasty" [Title/Abstract]) OR "Combined approach tympanoplasty" [Title/Abstract] OR CWU [Title/Abstract] OR CWD [Title/Abstract] OR CAT [Title/Abstract]) |
| Scopus | (TITLE-ABS-KEY ( "Otitis Media" OR "Chronic Otitis Media" OR "cholesteatoma" ) AND TITLE-ABS-KEY ( "Quality of Life" OR qol OR "Quality of Life" OR "Well-being" OR "Health-related quality of Life" ) AND TITLE-ABS-KEY ( "Otologic Surgical Interventions" OR "Otologic Surgical Procedures" OR "surgical treatment" OR tympanoplasty OR myringoplasty OR mastoidectomy OR "Mastoid obliteration" OR "Bony obliteration Tympanoplasty" OR "Canal Wall up" OR "Canal Wall Up Tympanoplasty" OR "Canal Wall Down" OR "Canal Wall Down Tympanoplasty" OR cwu OR cwd OR cat OR "combined approach tympanoplasty" )) |
| Web of Science | ((TS=("Otitis Media" OR "Chronic Otitis Media" OR cholesteatoma OR cholesteatoma)) AND (TS=("Quality of Life" OR QoL OR "Quality of Life" OR "Health-related quality of life" OR HRQoL OR "Well-being" ))) AND TS=((Tympanoplasty OR myringoplasty OR mastoidectomy OR "Mastoid obliteration" OR "Bony obliteration Tympanoplasty" OR "Canal Wall up" OR "Canal Wall Up Tympanoplasty" OR "Otologic Surgical Procedures" OR "Otologic Surgical Interventions" OR "Surgical Treatment" OR "Canal Wall Down" OR "Canal Wall Down Tympanoplasty" OR "Combined approach tympanoplasty" OR CWU OR CWD OR CAT )) |
| Embase | (exp chronic otitis media/ OR "chronic otitis media" or "otitis media".ab,kf,ot,ti. OR cholesteatoma.ab,kf,ot,ti. OR exp cholesteatoma/) AND (exp "quality of life"/ OR "quality of life".ab,kf,ot,ti. OR QoL.ab,kf,ot,ti. OR "Health-related quality of life".ab,kf,ot,ti. OR "Well-being".ab,kf,ot,ti. OR HRQoL.ab,kf,ot,ti.) AND (exp tympanoplasty/ OR exp ear surgery/ OR exp myringoplasty/ OR exp canal wall up mastoidectomy/ OR exp canal wall down mastoidectomy/ OR exp mastoidectomy/ OR "Otologic Surgical Intervention".ab,kf,ot,ti. OR "Otologic Surgical procedure".ab,kf,ot,ti. OR "Surgical treatment".ab,kf,ot,ti. OR tympanoplasty.ab,kf,ot,ti. OR myringoplasty.ab,kf,ot,ti. OR "Mastoid obliteration".ab,kf,ot,ti. "Bony obliteration Tympanoplasty".ab,kf,ot,ti. OR "Canal wall up".ab,kf,ot,ti. OR "Canal wall down".ab,kf,ot,ti. OR "Canal wall up tympanoplasty".ab,kf,ot,ti. OR "Canal wall down tympanoplasty".ab,kf,ot,ti. OR "Combined approach tympanoplasty".ab,kf,ot,ti. OR CWU.ab,kf,ot,ti. OR  CWD.ab,kf,ot,ti. OR CAT.ab,kf,ot,ti.) |
| PubMed *initial orienting search November 2022* | ("Otitis Media" [Mesh] OR "Chronic Otitis Media" [Title/Abstract]) AND ("Quality of Life"[Mesh] OR QoL [Title/Abstract] OR "Quality of Life" [Title/Abstract] OR "Well-being" [Title/Abstract]) |

| ***Supplementary Table S3: Study quality and risk of bias assessment with NHLBI assessment tool for before-after [pre-post] studies without control group*** | | | | | | | | | | | | | | | |
| --- | --- | --- | --- | --- | --- | --- | --- | --- | --- | --- | --- | --- | --- | --- | --- |
| **Authors (ref)** | **1** | **2** | **3** | **4** | **5** | **6** | **7** | **8** | **9** | **10** | **11** | **12** | **13** | **Risk of Bias** | **Overall study quality** |
| **Baumann et al. (8)** | Yes | Yes | Yes | NR | NR | Yes | Yes | NR | No | No | Yes | Yes | No | **Low** | **Fair** |
| **Lailach et al. (31)** | Yes | Yes | Yes | No | Yes | Yes | Yes | No | No | No | Yes | Yes | No | **Low** | **Good** |
| **Nallapaneni et al. (32)** | Yes | Yes | No | Yes | Yes | Yes | Yes | NR | Yes | No | Yes | Yes | No | **Low** | **Good** |
| **Cavalier et al. (33)** | Yes | Yes | Yes | NR | CD | Yes | Yes | NR | NR | NR | Yes | Yes | No | **Low** | **Good** |
| **Nurmukhamedova et al. (34)** | Yes | No | No | CD | CD | CD | Yes | NR | CD | NR | Yes | Yes | No | **High** | **Poor** |
| **Choi et al. (35)** | Yes | Yes | Yes | No | NR | No | Yes | NR | Yes | No | Yes | Yes | No | **Low** | **Good** |
| **Nadol et al. (3)** | No | No | Yes | NR | NR | No | Yes | NR | No | No | Yes | No | No | **High** | **Poor** |
| **Jung et al. (36)** | Yes | No | Yes | NR | NR | No | Yes | NR | Yes | No | Yes | Yes | No | **High** | **Poor** |
| **Lucidi et al. (37)** | Yes | Yes | Yes | Yes | NR | Yes | Yes | NR | No | No | Yes | Yes | No | **Low** | **Fair** |
| **Nair et al. (38)** | Yes | Yes | Yes | NR | CD | Yes | Yes | NR | CD | NR | Yes | Yes | No | **High** | **Fair** |
| **Bächinger et al. (39)** | Yes | Yes | Yes | Yes | CD | No | Yes | NR | Yes | No | Yes | Yes | No | **Low** | **Fair** |
| **Bächinger et al. (40)** | Yes | No | Yes | NR | NR | No | Yes | NR | NR | NR | Yes | Yes | No | **High** | **Poor** |
| **Weiss et al. (41)** | Yes | No | Yes | CD | CD | Yes | Yes | NR | No | Yes | Yes | Yes | No | **High** | **Fair** |
| **Tailor et al. (42)** | Yes | Yes | Yes | Yes | NR | No | Yes | NR | Yes | Yes | Yes | Yes | No | **Low** | **Fair** |
| **Bukurov et al. (43)** | No | No | Yes | NR | Yes | Yes | Yes | NR | Yes | Yes | Yes | No | No | **Low** | **Fair** |
| **Baetens et al. (44)** | Yes | Yes | Yes | NR | Yes | Yes | Yes | No | CD | NA | Yes | Yes | No | **Low** | **Good** |
| *CD = cannot determine, NR = Not Reported, NA = Not Applicable*  *Items:* ***1.*** *Was the study question or objective clearly stated;* ***2.*** *Were eligibility/selection criteria for the study population prespecified and clearly described?;* ***3.***  *Were the participants in the study representative of those who would be eligible for the test/service/intervention in the general or clinical population of interest?;* ***4.***  *Were all eligible participants that met the prespecified entry criteria enrolled?.* ***5.*** *Was the sample size sufficiently large to provide confidence in the findings?;* ***6.*** *Was the test/service/intervention clearly described and delivered consistently across the study population?;* ***7.*** *Where the outcome measures prespecified, clearly defined, valid, reliable, and assessed consistently across all study participants?;* ***8.*** *Where the people assessing the outcomes blinded to the participants' exposures/interventions?;* ***9.*** *Was the loss to follow-up after baseline 20% or less?;* ***10.*** *Where those lost to follow-up accounted for in the analysis?;* ***11.*** *Did the statistical methods examine changes in outcome measures from before to after the intervention?;* ***12.*** *Were statistical tests done that provided p values for the pre-to-post changes?;* ***13.****Where outcome measures of interest taken multiple times before the intervention and multiple times after the intervention (i.e., did they use an interrupted time-series design)?* (26) | | | | | | | | | | | | | | | |
